# SIRT1 attenuates sepsis-induced acute kidney injury via Beclin1 deacetylation-mediated autophagy activation

**DOI:** 10.1038/s41419-021-03508-y

**Published:** 2021-02-26

**Authors:** Zhiya Deng, Maomao Sun, Jie Wu, Haihong Fang, Shumin Cai, Sheng An, Qiaobing Huang, Zhenfeng Chen, Chenglun Wu, Ziwei Zhou, Haoran Hu, Zhenhua Zeng

**Affiliations:** 1 Department of Critical Care Medicine, Nanfang Hospital, Southern Medical University, Baiyun District, Guangzhou, Guangdong 510515 China; 2 Guangdong Provincial Key Laboratory of Shock and Microcirculation, School of Basic Medical Sciences, Southern Medical University, Baiyun District, Guangzhou, Guangdong 510515 China; 3 Department of Anesthesiology, Nanfang Hospital, Southern Medical University, Baiyun District, Guangzhou, Guangdong 510515 China

**Keywords:** Macroautophagy, Acetylation

## Abstract

Our previous studies showed that silent mating-type information regulation 2 homologue-1 (SIRT1, a deacetylase) upregulation could attenuate sepsis-induced acute kidney injury (SAKI). Upregulated SIRT1 can deacetylate certain autophagy-related proteins (Beclin1, Atg5, Atg7 and LC3) in vitro. However, it remains unclear whether the beneficial effect of SIRT1 is related to autophagy induction and the underlying mechanism of this effect is also unknown. In the present study, caecal ligation and puncture (CLP)-induced mice, and an LPS-challenged HK-2 cell line were established to mimic a SAKI animal model and a SAKI cell model, respectively. Our results demonstrated that SIRT1 activation promoted autophagy and attenuated SAKI. SIRT1 deacetylated only Beclin1 but not the other autophagy-related proteins in SAKI. SIRT1-induced autophagy and its protective effect against SAKI were mediated by the deacetylation of Beclin1 at K430 and K437. Moreover, two SIRT1 activators, resveratrol and polydatin, attenuated SAKI in CLP-induced septic mice. Our study was the first to demonstrate the important role of SIRT1-induced Beclin1 deacetylation in autophagy and its protective effect against SAKI. These findings suggest that pharmacologic induction of autophagy via SIRT1-mediated Beclin1 deacetylation may be a promising therapeutic approach for future SAKI treatment.

## Introduction

Sepsis is associated with physiological, pathological and biochemical abnormalities induced by infection^1^. Sepsis-induced acute kidney injury (SAKI) is a common and severe complication in septic patients and often contributes to high morbidity and mortality^2^. Moreover, SAKI survivors have an increased prevalence of chronic kidney disease^3^. At present, there is no ideal therapy for SAKI and effective treatments are urgently needed. Thus, it is of priority to fully understand the pathogenesis of SAKI.

Recent studies have confirmed that autophagy, a self-degradative process in cells depending on lysosomes, exerts protective effects against SAKI^4^. Autophagy and its associated pathways are potential targets and therapeutic interventions for SAKI. In a murine model of SAKI, diminished autophagy leads to failed recovery of renal function^5^. In contrast, autophagy activation has a protective effect in SAKI^6^. An important autophagy protein, Beclin1, can form different complexes that are involved in autophagosome initiation and autolysosome maturation^7^. Beclin1-dependent autophagy protects multiple organs, such as the heart, lung and liver, during the pathogenesis of sepsis^8,9^. Considerable evidence also suggested that multiple autophagy genes (Atg) are required for the formation of autophagosomes and the subsequent induction of autophagy^10^. The expression of autophagy proteins, including Atg5, Atg7 and LC3, was decreased in the kidneys after lipopolysaccharide (LPS) challenge in mice^11^. Emerging evidence shows that the activity of autophagy-related proteins (Beclin1, Atg5, Atg7 and LC3) is regulated by acetylation/deacetylation^10,12,13^.

Silent mating-type information regulation 2 homologue-1 (SIRT1) is an NAD^+^-dependent protein deacetylase that can deacetylate histone and nonhistone proteins. Our previous study showed that SIRT1 activation promotes HMGB1 deacetylation and thus attenuates inflammation and SAKI^14^. However, the exact protective mechanisms of SIRT1 in SAKI have not been fully clarified. Recent research has confirmed that SIRT1 can deacetylate Beclin1^13^, and thus activate Beclin1 and promote autophagy^15^, thereby protecting against traumatic brain injury^16^ and Alzheimer’s disease^17^. SIRT1 also deacetylates other autophagy-related proteins (Atg5, Atg7 and LC3) and thereby promotes autophagy and cell and tissue repair in response to nutrient starvation^10,12^. Thus, we hypothesise that SIRT1-deacetylated autophagy-related proteins enhance autophagy and thereby alleviate SAKI. The deacetylation of autophagy-related proteins to mediate autophagy induction may be a promising therapeutic approach for SAKI treatment. This study aims to investigate the role of SIRT1-mediated deacetylation of autophagy-related proteins in SAKI and underlying mechanisms.

## Results

### Dynamic changes in autophagy in an animal model of SAKI

**Fig. 1:**
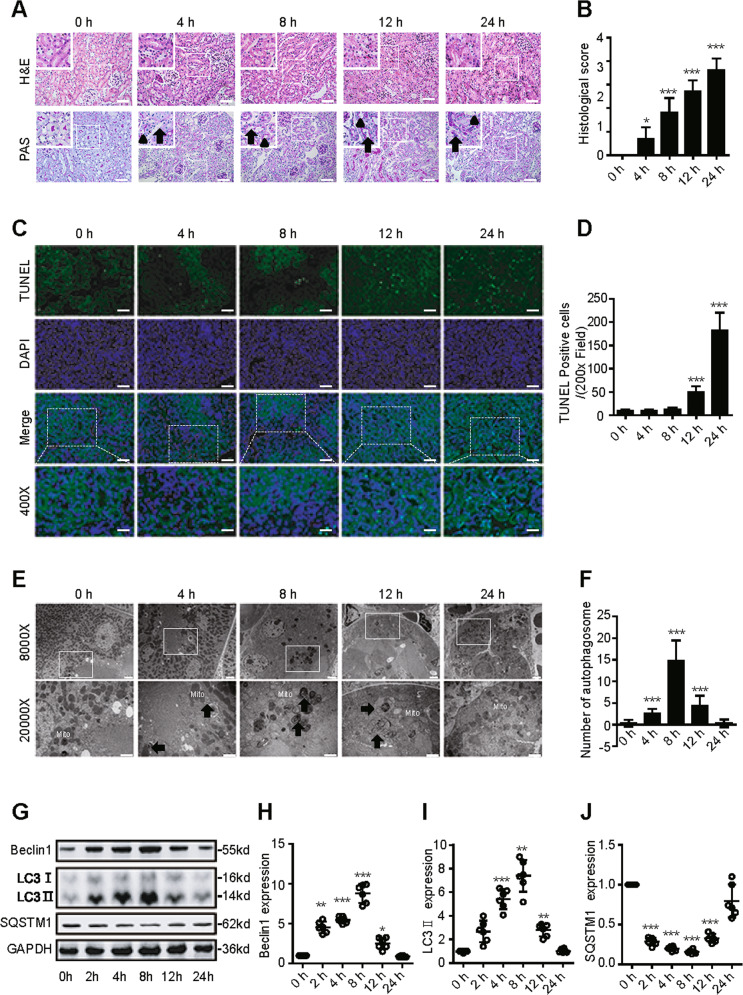
Dynamic changes in autophagy in an animal model of SAKI. **A** Pathological observation of the kidney cortex after H&E staining (upper panel) and PAS staining (lower panel). The irregular brush border (arrow) and ectasia of affected tubules (arrowheads) were observed following CLP surgery (upper panel: 200×; inset: 400×; scale bar = 10 μm). **B** The tubular damage score was evaluated based on pathological observation (*n* = 10). **C** Apoptosis was semi-quantitatively analysed by TUNEL-positive cell staining (upper panel: 200×; lower panel: 400×; scale bar = 5 μm). **D** The number of TUNEL-positive cells (*n* = 10). **E** Morphological observation of autophagy in the kidney cortex under an electron microscope (black arrow: autophagosomes; Mito: mitochondrion; upper panel: magnified ×8000 and scale bar = 2 μm; lower panel: magnified ×20000 and scale bar = 1 μm). **F** The number of autophagosomes in renal epithelial cells was calculated in 20 randomly selected fields (*n* = 20). **G**–**J** Representative western blot with densitometric analysis of Beclin1, LC3 II and SQSTM1 protein expression (*n* = 6). The data are presented as the mean ± SD. ^*^p < 0.05, ^**^p < 0.01 and ^***^p < 0.001 vs the 0-h group. SAKI sepsis-induced acute kidney injury, CLP caecal ligation and puncture, PAS periodic acid–Schiff staining, H&E haematoxylin–eosin staining, TUNEL terminal deoxynucleotidyl transferase dUTP nick end labelling staining, GAPDH glyceraldehyde 3-phosphate dehydrogenase.

First, we determined whether caecal ligation and puncture (CLP)-induced SAKI was successfully established in mice. Histopathological techniques such as haematoxylin-eosin (H&E) staining, periodic acid-Schiff (PAS) staining and transferase dUTP nick end labelling (TUNEL)-positive cell staining were applied. Consistent with previous studies^18^, renal tubular epithelial cell (RTEC) injury mainly presented as tubular dilatation, epithelial flattening and cell sloughing 4 h after CLP. The amount of tubular damage was more than doubled at 8 h and increased by 75% at 12 h, and severe coagulative necrosis occurred at 24 h after CLP (Fig. 1A, B). An increased level of TUNEL-positive RTECs was observed from 12 to 24 h after CLP (Fig. 1C, D). Interestingly, the number of autophagosomes gradually increased at 4 h, peaked at 8 h and then decreased (Fig. 1E, F). Western blotting showed that the expression of the autophagy-related proteins Beclin1 and LC3 increased after CLP, gradually peaked at 8 h, and returned to baseline by 24 h (Fig. 1G–I). In contrast, sequestosome 1 (SQSTM1, a mediator of cargo selection and an autophagic substrate) expression reached the lowest levels at 8 h after CLP, which was consistent with the autophagosomes analysis (Fig. 1G, J). These results indicated that autophagy is temporarily activated and then continues to decline in SAKI. Therefore, 12 h after CLP was selected as the time point for further study.

### Activation of autophagy protects against SAKI in RTECs

**Fig. 2:**
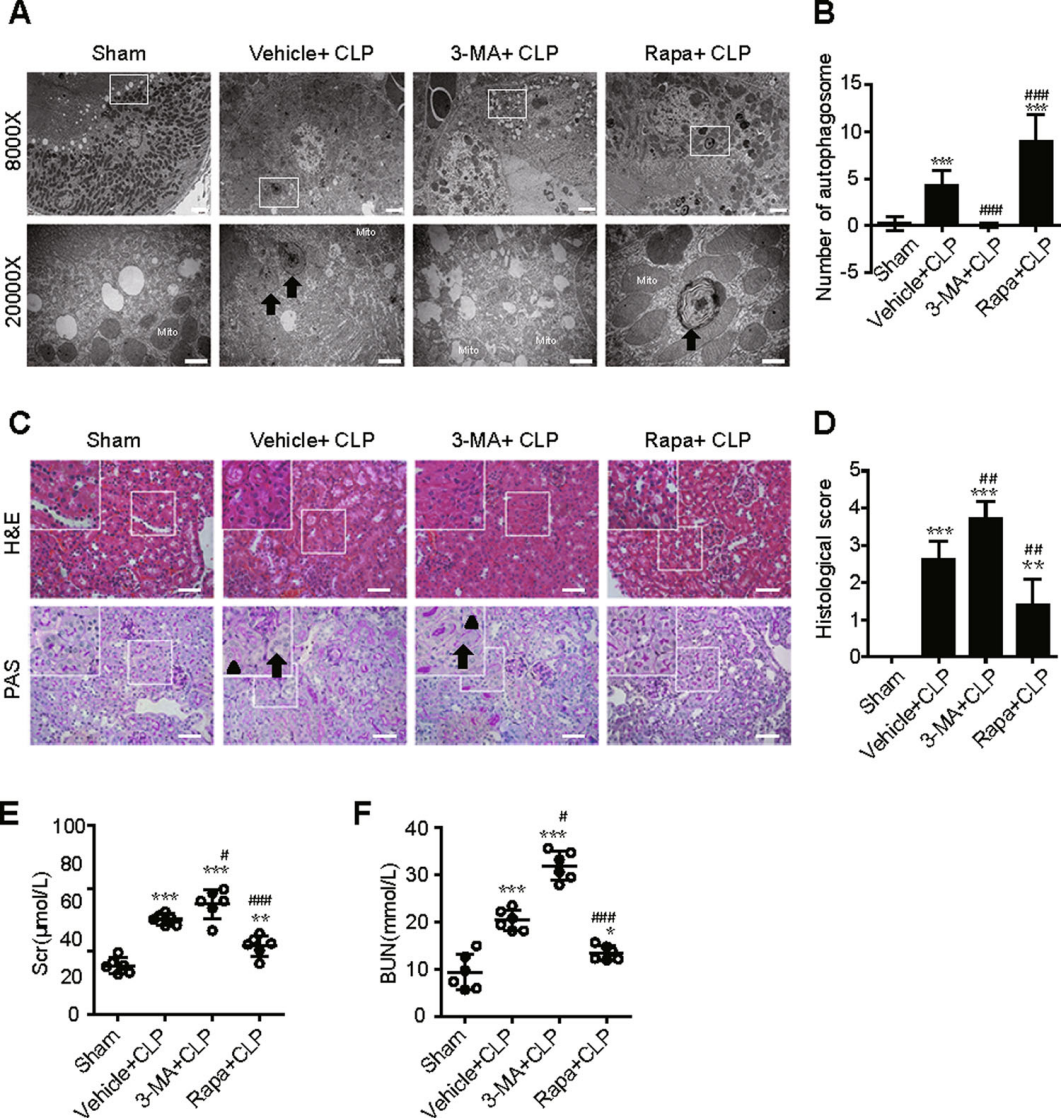
Activation of autophagy protects against SAKI in RTECs. Autophagy activation or inhibition by pretreatment with Rapa or 3-MA, respectively. The samples were collected 12 h after CLP-induced SAKI. **A**, **B** The number of autophagosomes in renal epithelial cells was calculated in 20 randomly selected fields using a transmission electron microscope (black arrow: autophagosomes; Mito: mitochondrion; upper panel: magnified ×8000 and scale bar = 2 μm; lower panel: magnified ×20000 and scale bar = 1 μm, *n* = 20). **C** The irregular brush border (arrow) and ectasia of affected tubules (arrowheads) were observed by H&E staining (upper panel, 200×; inset: 400×; scale bar = 10 μm) and PAS staining (lower panel, 200×; inset: 400×; scale bar = 10 μm) of the kidney cortex. **D** The tubular damage score was evaluated based on H&E and PAS staining (*n* = 10). **E** Scr levels (*n* = 6). **F** Serum BUN levels (*n* = 6). The data are presented as the mean ± SD. ^*^p < 0.05, ^**^p < 0.01 and ^***^p < 0.001 vs the sham group; ^*#*^*p* < 0.05, ^*##*^*p* < 0.01 and ^*###*^*p* < 0.001 vs the vehicle + CLP group. RTECs renal tubular epithelial cells, SAKI sepsis-induced acute kidney injury, CLP caecal ligation and puncture, PAS periodic acid-Schiff staining, H&E haematoxylin–eosin staining, Rapa rapamycin, Scr serum creatinine, BUN urea nitrogen.

To investigate the exact role of autophagy in SAKI, the autophagy agonist rapamycin (Rapa) and the autophagy inhibitor 3-MA were used. Autophagy activation by Rapa administration attenuated CLP-induced hydropic mitochondria with damaged cristae and loss of the microvillus brush border with damaged apical membranes in proximal tubules, as well as a decreased number of autophagosomes (Fig. 2A, B). In contrast, autophagy inhibition by 3-MA significantly exacerbated CLP-elicited alterations (Fig. 2A, B). Rapa administration also attenuated SAKI induced by CLP, as evidenced by reduced renal histological scores (Fig. 2C, D) and blood urea nitrogen (BUN) and serum creatinine (Scr) levels (Fig. 2E, F). In contrast, autophagy inhibition by 3-MA exacerbated CLP-induced SAKI (Fig. 2C–F).

### SIRT1 attenuates SAKI by promoting autophagy

**Fig. 3:**
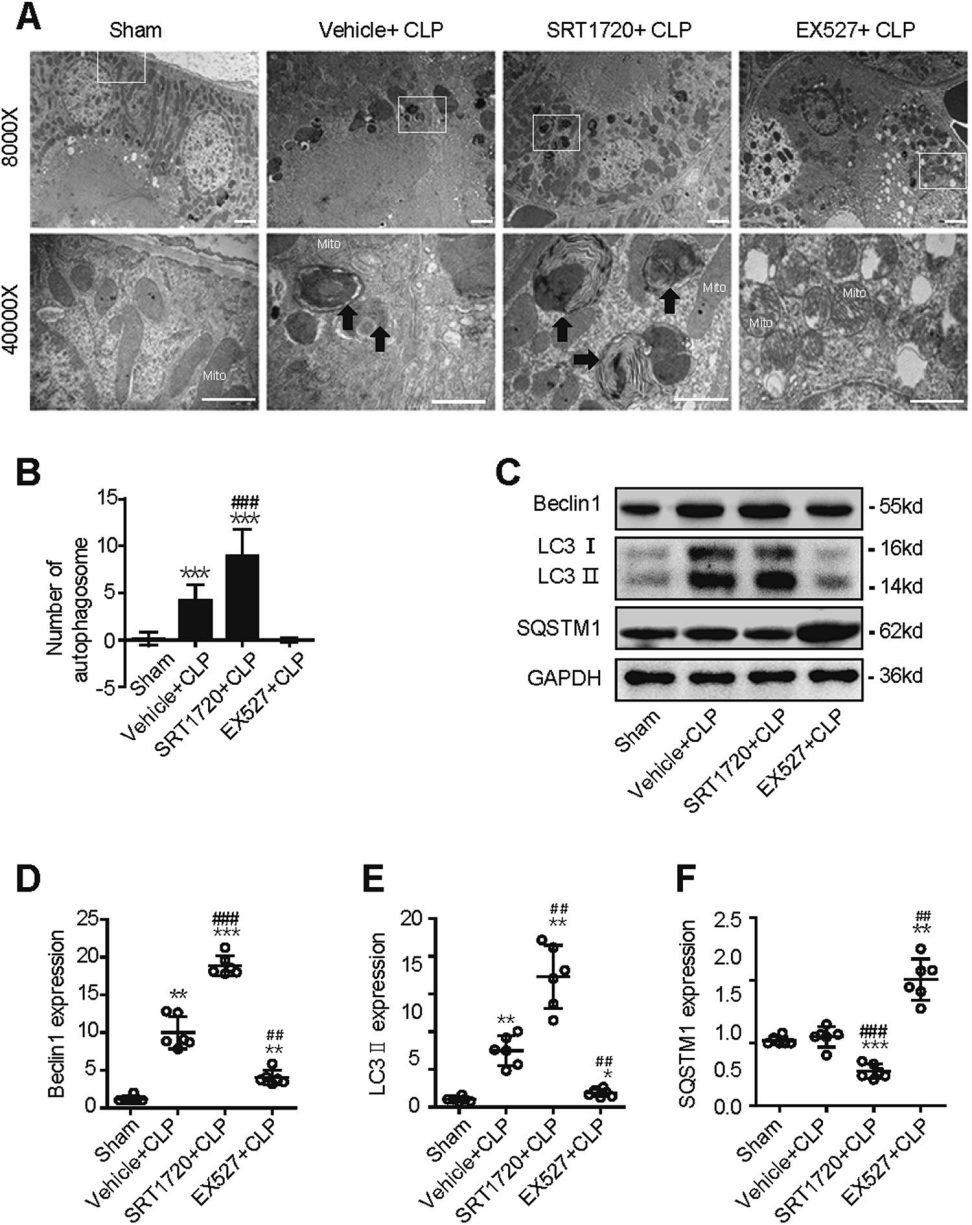
SIRT1 attenuates SAKI by promoting autophagy. Mice was pretreated with SRT1720 or Ex527 for 2 h before CLP surgery to activate or inhibit SIRT1, respectively. Kidney tissue samples were collected 12 h after CLP surgery. **A**, **B** The kidney cortex was observed and the number of autophagosomes was calculated in 20 randomly selected fields using a transmission electron microscope (black arrow: autophagosomes; Mito: mitochondrion; upper panel: magnified ×8000 and scale bar = 2 μm; lower panel: magnified ×40000 and scale bar = 1 μm, *n* = 20). **C**–**F** Representative western blot and densitometric analysis of Beclin1, LC3 II and SQSTM1 protein expression (*n* = 6). The data are presented as the mean ± SD. ^*^p < 0.05, ^**^p < 0.01 and ^***^p < 0.001 vs the sham group; ^*##*^*p* < 0.01, ^*###*^*p* < 0.001 vs the vehicle + CLP group. SAKI sepsis-induced acute kidney injury, CLP caecal ligation and puncture, SQSTM1 sequestosome 1, GAPDH glyceraldehyde 3-phosphate dehydrogenase.

Next, we investigated whether SIRT1 attenuates SAKI by promoting autophagy. The selective SIRT1 agonist SRT1720 and the selective SIRT1 inhibitor Ex527 were used in an animal model of SAKI. SIRT1 activation by SRT1720 attenuated mitochondria swelling characterised by damaged cristae, the loss of the microvillus brush border and increased number of autophagosomes in SAKI. SIRT1 inhibition by Ex527 exacerbated CLP-induced mitochondria swelling and led to reduced formation of autophagosomes (Fig. 3A, B). Western blotting showed that the protein expression of Beclin1 and LC3 II was increased after SIRT1 activation by SRT1720 and decreased after SIRT1 inhibition by Ex527 (Fig. 3C–E). In contrast, SQSTM1 expression showed the opposite trend. (Fig. 3C, F). These results indicate that SIRT1 attenuates SAKI by promoting autophagy.

### Dynamic changes in autophagy in a SAKI cell model

**Fig. 4:**
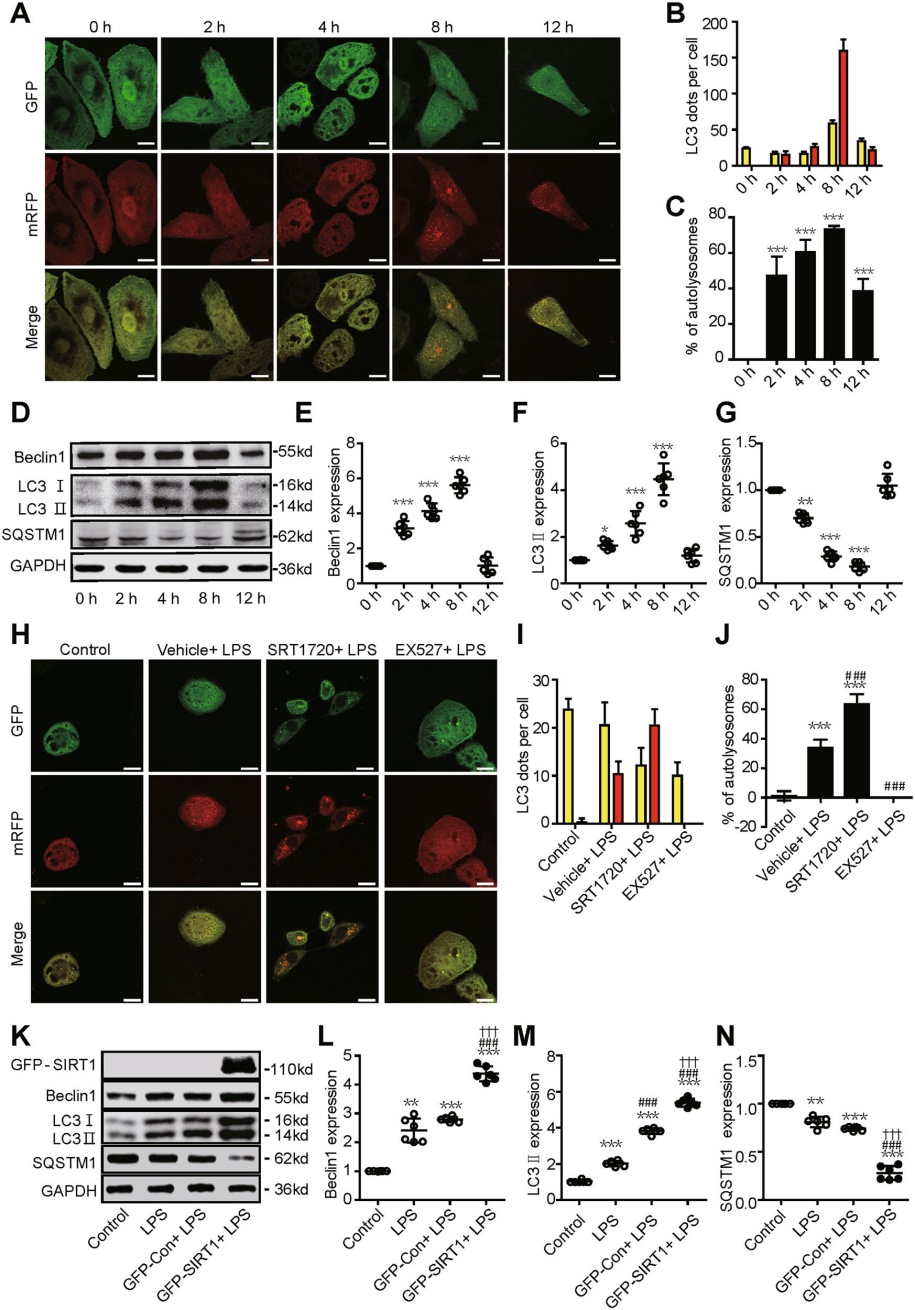
Dynamic changes in autophagy in a SAKI cell model. HK-2 cells were treated with different concentrations of LPS (2, 4, 8 and 12 h with 10 μg/ml). **A** Autophagy increased in a time-dependent manner following LPS challenge, peaked at 8 h and then gradually decreased. HK-2 cells were infected with an adenovirus expressing mRFP-GFP-LC3 (magnification ×630 and scale bar = 20 μm). **B** Mean numbers of autophagosomes (yellow dots per cell in merged images) and autolysosomes (red dots per cell in merged images) were calculated in 20 randomly selected cells (*n* = 20). **C** The percentage of autolysosomes (free red spots/(yellow spots+free red spots) per cell) was calculated in 20 randomly selected cells to determine autophagic flux (*n* = 20). **D**–**G** Representative western blots and densitometric analysis of Beclin1, LC3 II and SQSTM1 protein expression (*n* = 6). The data are presented as the mean ± SD. ^*^p < 0.05, ^**^p < 0.01 and ^***^p < 0.001 vs the 0-h group. **H** Autophagic flux determination and analysis: HK-2 cells were infected with an adenovirus expressing mRFP-GFP-LC3 (magnification ×630 and scale bar = 20 μm). **I** Mean numbers of autophagosomes (yellow dots per cell in merged images) and autolysosomes (red dots per cell in merged images) was calculated in 20 randomly selected cells (*n* = 20). **J** The percentage of autolysosomes (free red spots/(yellow spots + free red spots) per cell) were calculated in 20 randomly selected cells to determine autophagic flux (*n* = 20). SIRT1 activation or inhibition was performed by pretreatment with SRT1720 or EX527 for 2 h, respectively. Samples were collected 12 h after LPS stimulation. The data are presented as the mean ± SD. ^***^p < 0.001 vs the control group, ^*###*^*p* < 0.001 vs the vehicle + LPS group. **K**–**N** Representative western blots and densitometric analysis of the autophagy-related proteins Beclin1, LC3 II and SQSTM1 proteins (*n* = 6). The data are presented as the mean ± SD. ^**^p < 0.01 and ^***^p < 0.001 vs the control group; ^*###*^*p* < 0.001 vs the LPS group; ^*†††*^*p* < 0.001 vs the GFP-Con + LPS group. SAKI sepsis-induced acute kidney injury, LPS lipopolysaccharide, SQSTM1 sequestosome 1, SIRT1 silent mating-type information regulation 2 homologue-1, GAPDH glyceraldehyde 3-phosphate dehydrogenase.

We next established an LPS-treated HK-2 cell model of SAKI to investigate the molecular mechanisms in the pathogenesis of SAKI. Consistent with the animal model results, the autophagic flux in HK-2 cells was transiently increased in response to LPS stimulation, peaked at 8 h, and then gradually decreased to baseline levels (Fig. 4A–C). Accordingly, the protein expression of Beclin1 and LC3 II rapidly increased at first and then slightly decreased (Fig. 4D–F) and the protein expression of SQSTM1 showed the opposite trend (Fig. 4D, G).

Consistently, SIRT1 activation by SRT1720 increased autophagic flux, while SIRT1 inhibition by Ex527 decreased autophagic flux (Fig. 4H–J). Furthermore, we overexpressed SIRT1 with a plasmid vector in HK-2 cells to confirm the role of SIRT1. Further western blotting results showed that SIRT1 overexpression had the same role in the regulation of protein expression of the autophagy-related protein (Beclin1, LC3 II and SQSTM1) (Fig. 4K–N).

### SIRT1 activation promotes the deacetylation of Beclin1 but not the other autophagy-related proteins

**Fig. 5:**
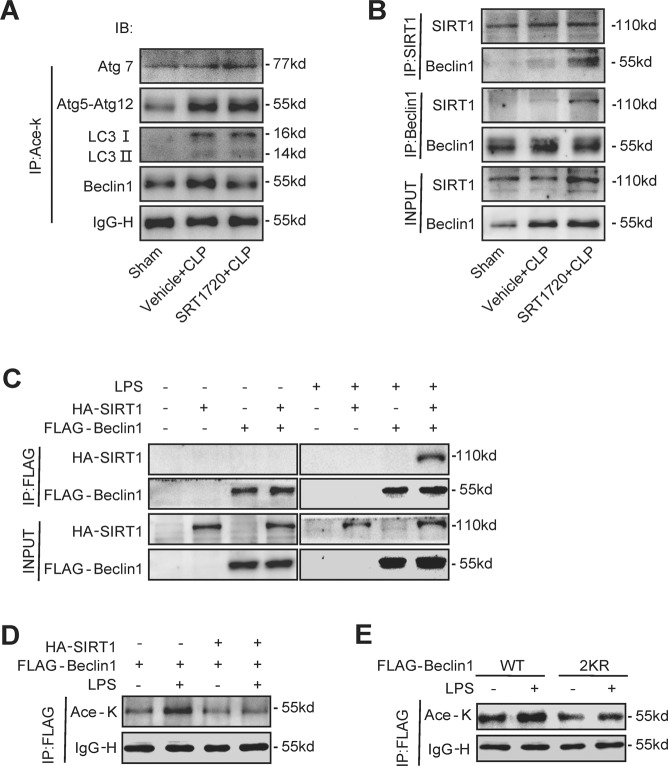
SIRT1 activation promotes the deacetylation of Beclin1 but not the other autophagy-related proteins. The mice were euthanized 12 h after CLP-induced SAKI. **A** The acetylation levels of Atg5–Atg12, Atg7, LC3 and Beclin1 and representative western blots are shown. The kidney samples were immunoprecipitated with anti-acetyl-lysine antibodies to analyse the acetylation of Atg5–Atg12, Atg7, LC3 and Beclin1. **B** Association of Beclin1 with SIRT1 in the SAKI animal model. The interaction between Beclin1 and SIRT1 was determined by Co-IP. **C** Association of Beclin1 with SIRT1 in the SAKI cell model. HA-tagged SIRT1 and FLAG-tagged Beclin1 were individually transfected into HK-2 cells with or without LPS stimulation. The interaction between Beclin1 and SIRT1 was determined by Co-IP. **D** The acetylation of Beclin1 after SIRT1 overexpression was examined by immunoprecipitation and western blotting. **E** The acetylation of Beclin1 markedly decreased after double mutation of K430 and K437. FLAG-tagged Beclin1 (WT, 2KR) was transfected into HK-2 cells with or without LPS stimulation. The acetylation of Beclin1 was examined in immunoprecipitation assays and representative western blots are shown. SAKI sepsis-induced acute kidney injury, LPS lipopolysaccharide, Co-IP Co-immunoprecipitation, SIRT1 silent mating-type information regulation 2 homologue-1.

Next, we investigated the effect of SIRT1 activation on the deacetylation of autophagy-related proteins (Atg5, Atg7, LC3 and Beclin1) during SAKI. The results showed that SIRT1 activation deacetylated Beclin1 but not the other autophagy-associated proteins (Fig. 5A). Therefore, we speculated that SIRT1 may induce autophagy in SAKI by directly deacetylating Beclin1. Therefore, Co-IP experiments were performed in both animal and cell models of SAKI. The results showed that SIRT1 directly interacted with Beclin1 when SIRT1 was activated by SRT1720 in SAKI animal model or was overexpressed with a plasmid in SAKI cell model (Fig. 5B, C). Intriguingly, we also found that SIRT1 did not interact with Beclin1 in the absence of LPS stimulation, indicating that this interaction may not occur in a steady state (Fig. 5C). Moreover, LPS treatment elevated the acetylation level of Beclin1, while SIRT1 overexpression markedly reduced the acetylation of Beclin1 (Fig. 5D). The results indicated that SIRT1 directly interacts with and deacetylates Beclin1 in SAKI.

Previous study revealed that SIRT1 deacetylated Beclin1 at the lysine sites of K430 and K437 in 293T cells^13^. To confirm whether SIRT1 deacetylates these exact sites of Beclin1 in SAKI^13^, we mutated the lysine (K430 and K437) to arginine (R) to mimic the SIRT1-mediated deacetylation of Beclin1. Beclin1 double mutation (2KR) abolished LPS-induced Beclin1 hyperacetylation (Fig. 5E).

### SIRT1 activates autophagy via the deacetylation of Beclin1

**Fig. 6:**
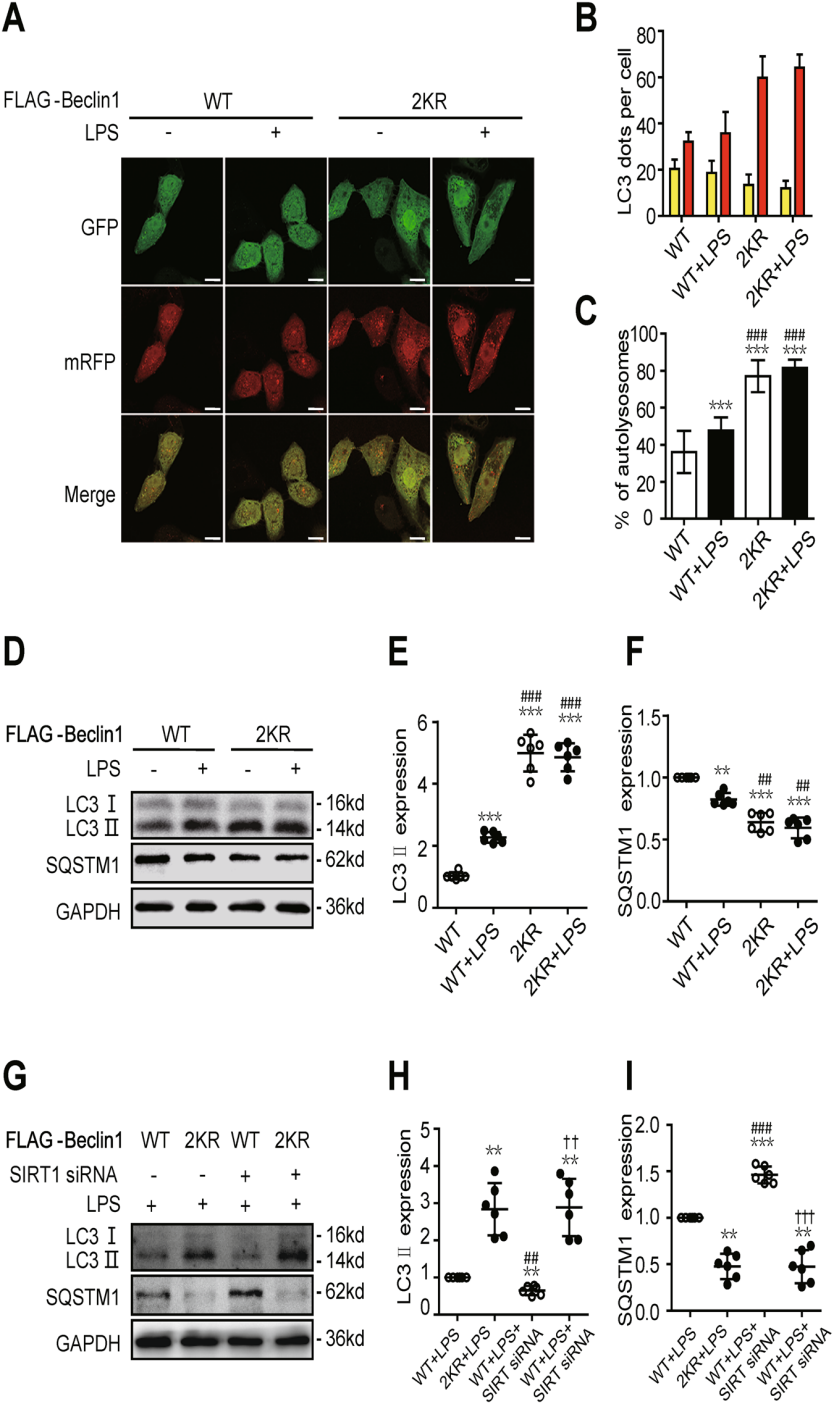
SIRT1 activates autophagy via the deacetylation of Beclin1. **A** In autophagic flux determination and analysis, HK-2 cells were infected with an adenovirus expressing mRFP-GFP-LC3 (magnification ×630 and scale bar = 20 μm). **B** Mean numbers of autophagosomes (yellow dots per cell in merged images) and autolysosomes (red dots per cell in merged images) were calculated in 20 randomly selected cells (*n* = 20). **C** The percentage of autolysosomes (free red spots/(yellow spots + free red spots) per cell) was calculated in 20 randomly selected cells to determine autophagic flux (*n* = 20). The data are presented as the mean ± SD. ^***^p < *0.001* vs WT Beclin1 over-expression control (WT group); ^*###*^*p* < *0.001* vs the WT + LPS group. **D**–**F** Representative western blots and densitometric analysis of the autophagy-related proteins Beclin1, LC3 II and SQSTM1 (*n* = 6). The data are presented as the mean ± SD. ^**^p < *0.01* and ^***^p < *0.001* vs the WT group; ^*##*^*p* < *0.01* and ^*###*^*p* < *0.001* vs the WT + LPS group. **G**–**I** Representative western blots of the autophagy-related proteins (LC3 II and SQSTM1) after SIRT1 knockdown or double mutation of Beclin1 (*n* = 6). The data are presented as the mean ± SD. ^**^p < *0.01* vs the WT + LPS group; ^*##*^*p* < *0.01* and ^*###*^*p* < *0.001* vs the 2KR + LPS group; ^*††*^*p* < 0.01 and ^*†††*^*p* < 0.001 vs the WT + LPS + SIRT1 siRNA group. SAKI sepsis-induced acute kidney injury, LPS lipopolysaccharide, SIRT1 silent mating-type information regulation 2 homologue-1, SQSTM1 sequestosome 1, GAPDH glyceraldehyde 3-phosphate dehydrogenase.

In SAKI cell model, double mutation of Beclin1 (2KR) increased autophagic flux and LC3 II expression, and decreased SQSTM1 expression, which mimicked the effect of SIRT1 activation (Fig. 6A–F). Rescue assays were performed in order to further confirm the role of SIRT1-mediated Beclin1 deacetylation in autophagy during the development of SAKI. Results showed that double mutation of Beclin1 (2KR) rescued SIRT1 knockdown-exacerbated LC3 II decrease and SQSTM1 increase (Fig. 6G–I). The results show that SIRT1 promotes autophagy by deacetylating the K430 and K437 sites of Beclin1 in SAKI.

### SIRT1 activation attenuates SAKI

**Fig. 7:**
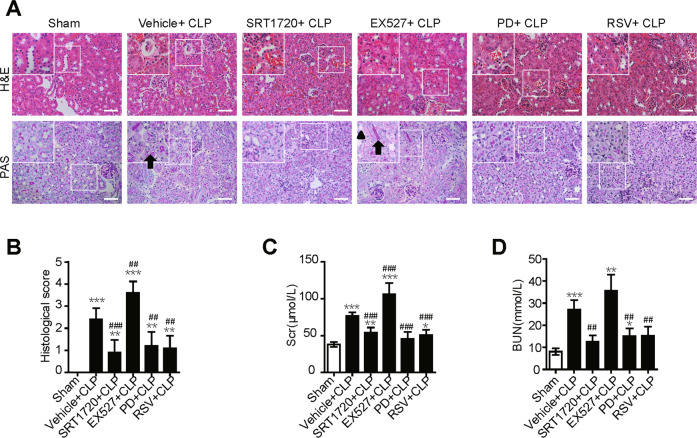
SIRT1 activation attenuates SAKI. SIRT1 activation by pretreatment with SRT1720, PD, RSV and SIRT1 inhibition pre-treated with EX527 for 2 h, respectively. **A** Pathological observation of kidney tissue. H&E staining of kidney cortex (upper panel, 200×; inset: 400×; scale bar = 10 μm) and PAS staining of kidney cortex (lower panel, 200×; inset: 400×; scale bar = 10 μm). The irregular brush border (arrow) and ectasia of the affected tubules (arrowheads) was observed. **B** Tubular damage scores were evaluated based on H&E and PAS staining (*n* = 10). **C** Scr levels (*n* = 6). **D** Serum BUN levels (*n* = 6). Values shown are the mean ± SD. ^*^p < 0.05, ^**^p < 0.01 and ^***^p < 0.001 vs the sham group; ^*##*^*p* < 0.01 and ^*###*^*p* < 0.001 vs the vehicle + CLP group. SAKI sepsis-induced acute kidney injury, CLP caecal ligation and puncture, SIRT1 silent mating-type information regulation 2 homologue-1, PD polydatin, RSV resveratrol, PAS periodic acid-Schiff staining, H&E haematoxylin-eosin staining, Scr Serum creatinine, BUN urea nitrogen.

Furthermore, we validated the protective role of SIRT1 activation in kidney function in SAKI. Expectedly, SIRT1 activation by SRT1720 significantly reduced the kidney tubular damage score and the levels of serum BUN and Scr (Fig. 7). A similar therapeutic effect was also observed by the potential SIRT1 activator resveratrol (RSV) and polydatin (PD). In contrast, SIRT1 inhibition by Ex527 treatment worsened renal function (Fig. 7). Taken together, our results suggest that SIRT1-deacetylated Beclin1 enhances autophagy and thus protects against SAKI.

## Discussion

**Fig. 8:**
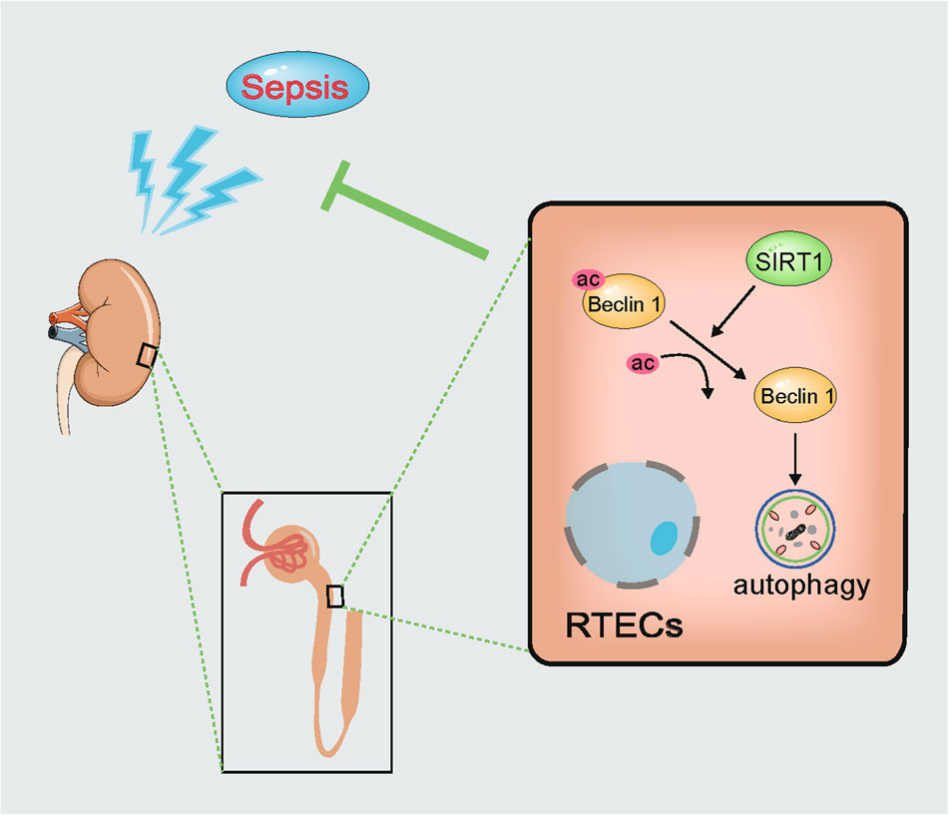
The effect of SIRT1-mediated Beclin1deacetylation induces autophagy in attenuating sepsis-induced acute kidney injury (SAKI). RTECs renal tubular epithelial cells, Ac acetylation.

In this study, we found that autophagy was transiently induced and then continued to decline during the development of SAKI, and that autophagy upregulation could attenuate SAKI. SIRT1 activation attenuated SAKI and was associated with autophagy upregulation. SIRT1 physically interacted with Beclin1 but not with other autophagy-related proteins (Agt5, Atg7 and LC3). SIRT1 directly deacetylated the K430 and K437 sites of the Beclin1 protein, which subsequently promoted autophagy and ameliorated SAKI. Our study emphasises that SIRT1-mediated deacetylation of Beclin1 activates autophagy and protects against SAKI (Fig. 8).

It is generally believed that the main pathogenic feature of SAKI is renal tubular damage rather than glomerular injury^19^. Therefore, this study focused on the renal cortex of septic mice and HK-2 cells. At present, abnormal autophagy, excessive apoptosis, severe mitochondrial damage, unbalanced inflammation and the adaptive response of RTECs have been reported in the pathogenesis of SAKI^20,21^. A previous postmortem study confirmed the presence of autophagosomes in RTECs in sepsis patients, suggesting that autophagy participates in the pathophysiological mechanism of SAKI^18^. Some studies suggest that excessive activation of autophagy worsens to SAKI^22^, but most studies have confirmed the benefits of autophagy in protecting against SAKI^23^. However, the exact role of autophagy in SAKI is not fully understood.

In this study, we confirmed that the autophagy level of RTECs temporarily increased in an early stage and then decreased markedly later in SAKI. Autophagy activation in RTECs after sepsis may be an adaptive protective mechanism. When autophagy decreased at 12 h after CLP-induced sepsis, cell apoptosis began to increase progressively. Therefore, we conducted subsequent experiments at 12 h after CLP-induced sepsis. Consistent with a previous study^23,24^, we also confirmed that drug-induced autophagy could attenuate proximal tubule dysfunction following sepsis in vivo. However, autophagy inhibition significantly exacerbated the renal injury. Our study showed that autophagy activation may be an effective therapeutic strategy for SAKI treatment.

Beclin1, an important autophagy effector, has been found to exert a protective effect in sepsis via activating autophagy^8,9^. At present, no effective agonist or chemical agent targeting Beclin1 has been investigated. Given that SIRT1-mediated deacetylation of autophagy proteins has a role in autophagy regulation, we examined the acetylation level of Beclin1 and the other autophagy-related proteins. An increased level of acetylation was observed in Beclin1, as well as in other autophagy-related proteins in SAKI. However, SIRT1 activation alleviated only Beclin1 hyperacetylation but not the other autophagy-related proteins. Further Co-IP assay confirmed the direct interaction between SIRT1 and Beclin1 in SAKI. Moreover, we proved that K430 and K437 in Beclin1 were target deacetylation sites of SIRT1. Although some other Beclin1-independent pathways might be involved in SAKI, those pathways may not be targeted by SIRT1. Beclin2, a homologue of Beclin1, acts as a regulator of autophagy in a Beclin1-independent pattern^25^. Its acetylation/deacetylation sites have not been identified and it requires more study to explore Beclin2-dependent pathway in SAKI and the relationship with SIRT1.

Considering that SIRT1 can deacetylate Beclin1 and activate autophagy, and that the natural polyphenol plant extracts polydatin and resveratrol which can activate SIRT1 was also used in the present study^26,27^. Prior studies and our previous research showed that SIRT1 was versatile in sepsis treatment and that drug-activated SIRT1 exerted multiple beneficial effects against sepsis^28^. We previously demonstrated that SIRT1 activation by polydatin or resveratrol protected against LPS-induced endothelial barrier dysfunction and AKI in sepsis^29,30^. Moreover, SIRT1 inhibited the translocation and secretion of HMGB1 by deacetylating HMGB1, attenuating sepsis-related liver^31^ and kidney^14^ injury and increasing the survival rate of septic mice^32^. In this study, we also demonstrated that SIRT1 agonists attenuated SAKI by Beclin1-dependent autophagy. We found two SIRT1 agonists, polydatin and resveratrol, attenuated SAKI and these findings may have potential clinical significance. Future clinical research is needed to fully validate the animal results in the present study.

Our study had some limitations. First, we did not monitor the dynamic acetylation levels of Beclin1 in SAKI because the acetylation of Beclin1 is easily saturated following sepsis induction. Second, we did not use Beclin1 + /- mice or Beclin1 kidney conditional knockout mice to further examine the relationship between SIRT1 and Beclin1 in vivo because mice that are homozygous for a Beclin1-knockout allele exhibit prenatal lethality, and additionally, our research mainly focused on the deacetylation of Beclin1. Third, due to some limitations, we do not have more clinical data to consolidate our findings. Although a study showed that injection of Tat-Beclin1 peptide to activate autophagy protects the heart during sepsis^8^, there are currently no effective clinical treatments for inducing the overexpression of Beclin1. We look forward to following-up clinical research to explore this topic.

In summary, we found that SIRT1 activation mitigates SAKI by promoting Beclin1-mediated autophagy. This study may provide new therapeutic targets for the treatment of SAKI.

## Materials and methods

### SAKI animal model study

Male C57BL/6 mice (7–8 weeks) weighing 18–22 g were acquired from the animal lab centre of Southern Medical University, Guangzhou, P.R. China. The animal experiments were conducted in accordance with the recommendations outlined in the Guidelines for the Care and Use of Laboratory Animals (National Institutes of Health, Bethesda, MD, USA) and the research programme was approved by the Ethics Committee of Animal Experiments of Southern Medical University. The sepsis model was established in mice using the classic CLP method^33^. The chemical reagents 4 mg/kg Rapa (S1039, Selleck) and 20 mg/kg 3-MA (S2767, Selleck) were intraperitoneally injected 2 h before CLP treatment. Ten milligram per kilogram SRT1720 (S1129, Selleck), 10 mg/kg EX527 (S1541, Selleck), 30 mg/kg RSV (S1396, Selleck) and 30 mg/kg PD (Neptunus Co. Ltd.) were injected via the tail vein 2 h before CLP treatment, respectively. The animals were divided into the following groups as required for the different experiments: (1) the sham group, (2) Vehicle + CLP group, (3) Rapa + CLP group, (4) 3-MA + CLP group, (5) SRT1720 + CLP group, (6) EX527 + CLP group, (7) PD + CLP group and (8) RSV + CLP group.

### SAKI cell model

HK-2 cells (human renal proximal tubular epithelial line, Kunming Cell Bank, Kunming, China) were cultured in DMEM/F12 supplemented with 10% (v/v) FBS at 37 °C in a humidified atmosphere containing 5% CO_2_. A recent mycoplasma contamination test conducted on HK-2 cells showed negative results. LPS (L2630, Sigma) was used to stimulate HK-2 cells to mimic SAKI in the cell model. According to the experimental groups, 10 μM Rapa, 5 mM 3-MA, 1 μM SRT1720 and 1 μM EX527 was added 2 h before LPS administration. The cells were transfected with empty plasmid vectors or HA-SIRT1 or GFP-SIRT1 plasmid vectors constructed using Lipofectamine 2000 (11668027, Invitrogen) for 6 h. The cells were used in experiments after transfection for 36 h. Cells were transfected with an adenoviral vector expressing FLAG-Beclin1 and FLAG-Beclin1 2KR (GeneChem Co. Ltd., Shanghai, China) and used in experiments after 48 h of transfection.

### Renal pathology and functional assessment

Renal pathology was assessed by the PAS and H&E methods. Briefly, renal tissues were sectioned, fixed with 10% formalin for approximately 24 h and then stained and observed under a light microscope (Olympus, Tokyo, Japan). According to previous studies, two pathologists who were blinded to the experiment scored tubular damage in ten fields per group at 200× magnification based on the percentage of cortical tubular necrosis: 0 = no damage, 1 = 1–25%, 2 = 26–50%, 3 = 51–75% and 4 = 76–100%^14,34^. The number of apoptosis-positive cells in ten randomly selected fields in the kidney tissue from each group was determined by the TUNEL assay^14^.

Renal function was assessed by measuring the levels of BUN and Scr. Blood samples were collected 12 h after CLP surgery, and then centrifuged at 3000 rpm for 15 min to separate the serum. BUN and Scr levels were measured using an automatic biochemical analyser (Chemray 240, Shenzhen, China).

### Ultrastructural observation with electron microscopy

Kidney tissues were cryosectioned in 2% formaldehyde and 2.5% glutaraldehyde in 0.1 M sodium cacodylate buffer for 1 h and then washed in PBS for 2 days. The samples were then fixed in 1% osmic acid, dehydrated with ethanol and acetone gradients, embedded in epoxy resin, and cut into ultrathin sections. The tissue sections were stained with uranyl acetate and lead citrate, and then observed under an H-7500 transmission electron microscope (Hitachi, Tokyo, Japan). The number of autophagosomes was counted in 20 randomly selected fields in each group at 8000× magnification^35^.

### Western blotting analysis

Kidney tissue and HK-2 cell extracts were treated with radioimmunoprecipitation assay RIPA lysis buffer containing 1× protease inhibitor cocktail. The proteins in the supernatants were separated using SDS-PAGE and transferred to polyvinylidene difluoride (PVDF) membranes. Nonspecific binding sites on the blots were blocked by incubation in 5% BSA for 1 h, and the membranes were then incubated overnight with primary antibodies and incubated with secondary antibodies for 1 h, the protein bands were visualised using enhanced chemiluminescence reagents. The primary antibodies used were anti-Beclin1 (11306-1-AP, Proteintech), anti-SQSTM1 (18420-1-AP, Proteintech), anti-LC3 (4108, CST), anti-GFP (AE012, ABclonal) and anti-GAPDH (AC002, ABclonal). The secondary antibodies used were HRP-conjugated anti-mouse (AS014, ABclonal) and anti-rabbit (AS003, ABclonal) antibodies. The band intensities were quantified using ImageJ software. The protein expression levels were standardised relative to the level of GAPDH.

### Adenoviral infection and autophagic flux analysis

HK-2 cells were infected with mRFP-GFP-LC3 adenovirus (Hanbio Co. Ltd., Shanghai, China) for 6 h. After mRFP-GFP-LC3 transfection for 48 h, a laser scanning confocal microscope (Zeiss LSM780, Thuringia, Germany) was used to confirm the transfection efficiency via the GFP fluorescence intensity. The expression of both mRFP and GFP in mRFP-GFP-LC3 tandem fluorescent proteins were used to track LC3. Increased numbers of free red spots/(yellow spots + free red spots) suggested elevated autolysosome formation, indicating increased autophagic flux. The number of spots of each colour was counted in 20 randomly selected cells in each group^35^.

### Immunoprecipitation and co-immunoprecipitation assays

The enriched protein was incubated with 2 μg of anti-acetyl lysine (A2391, ABclonal) or anti-FLAG (AE005, ABclonal) overnight at 4 °C. Next, 40 μl of protein A + G agarose beads (P2012, Beyotime) was added to the mixture and centrifuged at 4 °C for 3 h. The beads were eluted with SDS loading buffer. Western blotting was used to determine protein expression. The primary antibodies used were anti-Atg5 (YM3752, Immunoway), anti-Atg7 (A19604, ABclonal), anti-LC3, anti-Beclin1 (YM1326, Immunoway), anti-acetyl lysine, anti-HA (AE008, ABclonal) and anti-FLAG. The protein expression levels were standardised relative to the level of IgG-H.

### Statistical analysis

The results were analysed using one-way ANOVA with SPSS 20.0 software. The number of samples per group is indicated in the corresponding figure legends as *n*. A value of *p* < 0.05 was considered statistically significant, and all values are presented as the mean ± SD.
